# Asymmetry from an asymmetrical cannula interface and nasogastric tube during nasal high flow enhances dead-space clearance: a fluid dynamics study

**DOI:** 10.3389/fmed.2026.1823238

**Published:** 2026-05-29

**Authors:** Zane Goggin, Natalia Kabaliuk, Stanislav Tatkov

**Affiliations:** 1 Department of Mechanical Engineering, University of Canterbury, Christchurch, New Zealand; 2 Fisher & Paykel Healthcare Limited, Auckland, New Zealand

**Keywords:** asymmetrical, dead-space, finite volume analysis, nasal cannula, nasal high flow, nasogastric tube

## Abstract

**Background:**

During non-invasive respiratory support with nasal high flow (NHF), an asymmetrical interface (AI) featuring nasal prongs of different sizes has been shown to reduce re-breathing and improve gas exchange in hypoxemic and COPD patients. The objective of this study was to investigate the mechanisms of expired gas clearance during NHF in the presence of a nasogastric (NG) tube, which is commonly used post extubation and causes partial upper-airway occlusion.

**Methods:**

A computational fluid dynamics study was conducted using an averaged adult upper-airway model with breathing patterns representative of both healthy adults and patients with respiratory failure. Gas flow containing an end-tidal CO_2_ concentration of 5% was numerically simulated using Ansys Fluent (Ansys Inc., USA) during NHF at rates of 20 L/min and 60 L/min. The computational domain included the nasopharynx and the distal portions of commercially available nasal cannula interfaces.

**Results:**

During NHF, the AI splits the flow both inside and outside the prongs, resulting in differential pressure across the nasal cavities. At an NHF rate of 60 L/min this generated reverse flow through the choanae and increased gas clearance by more than 65%, with up to 91% of expired CO_2_ expelled through the less occluded naris, compared with a symmetrical cannula interface with similarly-sized nasal prongs. The position of the NG tube on the side of either the large or small prong had only a marginal effect on the almost complete clearance (100% vs. 98.6%) observed at an NHF rate of 60 L/min. However, at a lower NHF rate of 20 L/min, clearance increased from 29.6% to 53.1% when the NG tube was positioned on the side of the large prong.

**Conclusion:**

In the computational experiments, asymmetry in the upper-airway cross-sectional area produced by differences in nasal prong size and the presence of an NG tube during NHF alters the gas kinetics and may accelerate anatomical dead-space clearance, thereby reducing re-breathing of expired gas.

## Introduction

Nasal high flow (NHF) is an established form of non-invasive respiratory support in which heated, humidified air, with or without supplemental oxygen, is delivered to the patient via a loosely fitted nasal cannula interface at flow rates typically ranging from 20 to 60 L/min in adults (1). NHF is used for patients with respiratory failure in both acute and chronic care settings (2). NHF with supplemental oxygen was extensively used in severely hypoxemic patients during the peak years of the COVID-19 pandemic (3–5). In a recent randomized controlled trial, NHF demonstrated non-inferiority to non-invasive ventilation with respect to endotracheal intubation or death within 7 days in a broad population of patients with acute respiratory failure (ARF) (6).

In addition to its oxygenation efficiency, NHF induces physiological effects through two physical mechanisms that improve gas exchange and reduce the work of breathing: reduction of re-breathing of expired gas from the anatomical dead space in the upper airways and generation of positive airway pressure that dynamically varies with nasal breathing (7, 8). At the end of expiration, the anatomical dead space is filled with expired gas that is rich in carbon dioxide (CO_2_) and depleted in oxygen. Re-breathing of CO_2_ plays a key role in the control of respiration. In lung diseases such as pneumonia or COPD, physiological dead space is markedly increased and even a relatively small reduction of re-breathing achieved by purging expired gas from the upper airways during NHF may substantially improve gas exchange (8–11).

Dead-space clearance during NHF may be reduced at higher respiratory rates due to shortened expiratory time and a faster transition from expiration to inspiration (8). Increased expiratory resistance during NHF has been shown to reduce elevated respiratory rates by prolonging expiratory time, which may improve the efficiency of dead-space clearance in patients with tachypnea (7, 12). Improved clearance may enhance gas exchange, which may in turn slow breathing frequency (8).

Generation of positive airway pressure, like dead-space clearance, is dependent on NHF rate (8, 9, 13–16). NHF therapy typically uses a symmetrical cannula interface (SI) with two identically-sized prongs. Increased occlusion in both nares reduces leak around the prongs, elevating positive airway pressure due to increased resistance but reducing clearance efficiency (8). A recently introduced asymmetrical interface (AI), incorporating nasal prongs of different sizes, has been proposed to increase pressure during NHF while avoiding complete occlusion of the nares. The AI has been shown to further enhance respiratory support by NHF compared with the SI in COPD during exacerbation and hypoxemic patients with ARF. This effect is believed to be related to the improved dead-space clearance and arises from differential pressure between the nasal cavities, resulting in reverse flow through the choana at the end of expiration and faster clearance of expired gas (15, 17, 18). In recovering patients with normal breathing frequency who were extubated following surgery, trauma, or septic shock, NHF at a rate of 50 L/min with an AI did not affect gas exchange but improved comfort compared with an SI (19).

A nasogastric (NG) tube is commonly used to deliver or remove substances from the stomach but little is known how NG tubes may affect NHF therapy, particularly as they introduce asymmetry in the nasal passages. Recently, a retrospective study reported that the presence of an NG tube may be associated with NHF failure in children with bronchiolitis, providing a rationale for the current investigation (20). A bench-top study with a rigid upper-airway model demonstrated that the position and size of the NG tube produce a variable effect on the generated airway pressure and clearance of carbon dioxide (CO_2_) (21).

The anatomical complexity of the upper airways limits experimental investigation of NHF mechanisms within the nasal cavity. The objective of the study was to investigate nasal air-flow dynamics during NHF with an AI and NG using computational fluid dynamics (CFD), with a particular focus on the mechanism of expired CO_2_ clearance. The study tested a hypothesis that an NG tube like an AI creates asymmetry in the upper airway and affects clearance of dead space during NHF.

## Materials and methods

This deterministic CFD simulation study was designed to evaluate the mechanisms of dead-space clearance during NHF using an SI, as well as an AI and NG tube, both of which induce upper-airway asymmetry. All analyses were performed using a fixed adult upper-airway geometry under predefined boundary conditions. No human participants, physiological measurements, or clinical data were involved. For each configuration, the numerical solver produced a single, reproducible solution governed by the Navier–Stokes equations and the specified boundary conditions. Comparisons were made across systematically varied conditions to isolate the mechanistic effects of each factor.

Ansys Fluent 2023 R2 (Ansys Inc., USA) was the CFD solver to simulate breathing and NHF therapy with both an AI and SI at rates of 20 and 60 L/min. Simulations were run over a full breath with two different respiratory patterns used for each NHF rate. Additionally, a simulation with no therapy (natural nasal breathing) was performed for comparison. Simulations were also run to study the effect of an NG tube on CO_2_ clearance. With the AI, two sets of simulations were run: one with the NG tube in the cavity with the small prong and another with the NG tube in the cavity with the large prong. The post−processing was conducted in ANSYS CFD−Post.

### Computational domain

An averaged adult European upper-airway geometry was utilized to model the upper airway. The geometry was generated from computed tomography scans of 25 symptom-free adult Europeans (22). It encompassed both sides of the nasal cavity and extended to the nasopharynx. The geometry, smoother and more regularized than a typical airway due to averaging, retained all anatomical features of a normal airway.

**FIGURE 1:**
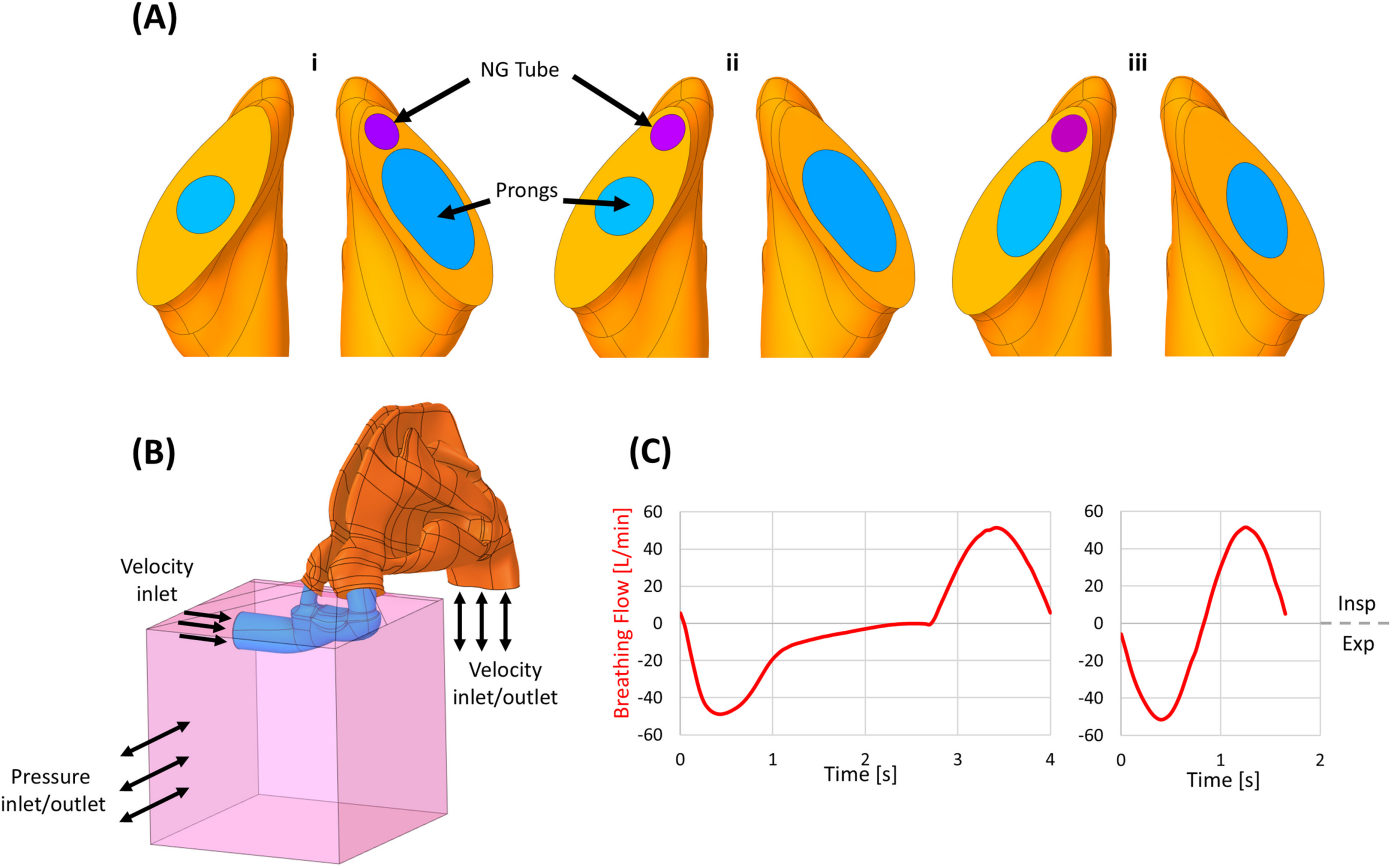
**(A)** Schematic of a nasogastric (NG) tube fitted into the nares with a symmetrical interface (SI) (iii), large prong cavity with an asymmetrical interface (AI) (ii) and small prong cavity (i) with an AI. **(B)** Model geometry showing nasal cavity (orange), asymmetrical interface (blue) and rectangular domain (pink). Velocity boundaries shown at the cannula inlet and nasopharynx. Pressure boundaries shown on a rectangular domain. **(C)** Breathing pattern with respiratory rates of 15 min^–1^ (left) and 35 min^–1^ (right).

The computational domain extended upstream of the nares, comprising a rectangular domain of dimensions 60 × 58 × 63 mm (Figure 1A). This setup enabled gas flow to enter the airway from various angles and facilitated the capture of gas previously expired from the airway (23, 24). The sizing of this domain was not verified through an independency test and therefore was chosen to be relatively large.

Both the AI and SI commercially available medium-sized cannulas were investigated (F&P Optiflow_TM_+ and Optiflow_TM_+ Duet, Fisher & Paykel Healthcare Limited, Auckland, New Zealand). The cannula geometry was created using the internal volume of the interfaces, extracted from the corresponding 3D models. The extracted geometries included the interface itself, and a section of the connecting tube cut off at the lateral end, as seen in Figure 1A. This provided a boundary for supplying the cannula flow (25). The cannula geometries were inserted into the airway model with approximately 60% of the prongs penetrating the airway. The cannula prongs are made from a soft material and can be deformed in order to fit into the nares. This deformability can be challenging to model in CFD, so the prongs were modeled as rigid but manually deformed to fit the shape of the nares by introducing greater curvature to both prongs. The larger prong of the AI also needed to also be slightly flattened to fit the oval shape of the nares. The airway was assumed to be rigid, and a no-slip boundary condition was applied, following similar CFD studies (24, 25). The AI creates asymmetrical occlusion in the nares (48.3% vs. 20.2%) due to the different cross-sectional area of the nasal prongs.

An 8 Fr (2.67 mm outer diameter) NG tube was modeled using a cylindrical tube. In one model, the tube was fitted into the nasal cavity with the large prong and, in another model, into the nasal cavity with the small prong. Another model was created with an NG tube and the SI cannula. The NG tube was inserted into the airway above the cannula prongs and extended through the nasal cavity to the nasopharynx boundary at the other end of the geometry. The positioning of the NG tube relative to the cannula in the nares is shown in Figure 1B.

### Meshing

The computational domain was discretized using the Watertight Geometry Workflow in Ansys Fluent Meshing (Ansys Inc., USA).

To accurately resolve near-wall flow behavior and capture the velocity gradients within the boundary layer, inflation layers were applied to the mesh on all airway walls. These layers ensure that the viscous sub-layer and transition to the logarithmic region are properly modeled, which is critical for predicting wall shear stress and pressure drop.

To correctly capture the velocity gradient at the no-slip boundary condition, five or three inflation layers with a growth rate of 1.1 were added to all airway walls. The first layer’s height was specified at 5E-05 m, keeping y+ values below 5. By default, in Ansys Fluent, all omega-based turbulence models have y+ insensitive wall treatment. The k-omega shear stress transport turbulence model was ultimately implemented, so a low y+ with highly refined boundary layers was not required (26).

A mesh sensitivity study was conducted to determine the number of cells needed. Using Richardson extrapolation, it was calculated that a mesh with 1.41E+06 cells would yield an acceptable error of about 0.8%. A poly-hex core mesh was chosen as it utilizes a combination of element types, resulting in greater efficiency than single element-type methods. Two buffer layers and one peel layer, with a maximum and minimum cell length of 2.24E-03 and 1.40E-04 respectively, were used to generate the mesh.xg150

### Boundary conditions

Three different boundary conditions were used within the domain. A no-slip wall boundary condition was applied to the airway walls and both the inside and outside of the cannula. A wall condition was also applied to the side of the rectangular domain representing the patient’s face. A pressure outlet was applied to the other five sides of this rectangular domain. A velocity inlet/outlet was applied to the cannula to supply NHF and at the nasopharynx to simulate breathing. Exact details on these boundary conditions can be found in the Ansys Fluent theory manual (26).

When modeling the SI, two velocity inlet boundaries were applied at the tips of the prongs, distributing the flow evenly between both prongs, in line with methodologies used in previous NHF CFD studies (23, 24, 27). Velocities of 8.0 and 24.0 m/s were applied to achieve total NHF rates of 20 and 60 L/min between the prongs. For the AI, the flow was expected to split unevenly between the two differently-sized prongs and to vary throughout the breathing cycle (15). Therefore, the flow in the interior of the AI was computed directly. To achieve this, a velocity inlet was applied at the lateral end of the cannula where the geometry was cut off (Figure 1A). Velocities of 4.1 and 12.3 m/s were applied to achieve NHF rates of 20 and 60 L/min at this inlet (Figure 1B).

For both the AI and SI cases, a velocity inlet/outlet was applied to the nasopharynx to simulate breathing. Two different breathing patterns were investigated (Figure 1C). These were a normal 15 min^–1^ breathing pattern of a healthy adult with an inspiration-to-expiration ratio of 1:2, and a 35 min^–1^ breathing pattern with equal inspiratory and expiratory times to represent the breathing pattern of a patient with ARF (15). Transient velocity profiles were applied to the nasopharynx boundary to match the flow rates.

### Solver settings

The model was solved isothermally, tracking two fluid species: humidified air at 100% relative humidity and CO_2_. In line with previous methodologies, a constant fraction of CO_2_ was applied to the nasopharynx boundary during exhalation to simulate expired gas (25). The volume fraction of CO_2_ in expired gas is approximately 4%–5%. A volume fraction of 5% was applied to the nasopharynx boundary. An atmospheric boundary condition of 0.04% CO_2_ volume fraction was applied at the pressure outlet around the rectangular domain and at the velocity inlet on the SI and AI cannulas. This simulated the case where no supplementary oxygen is added to the NHF gas. In agreement with other CFD NHF studies, a turbulence intensity of 5% was specified at all flow boundaries (24, 27).

To model turbulence, the Reynolds-Averaged Navier-Stokes Shear Stress Transport k-omega (RANS SST k-ω) model was used. A pressure-based solver with the Semi-Implicit Method for Pressure-Linked Equations (SIMPLE) pressure velocity coupling scheme and the Rhie-Chow distance-based flux approach was also used in all simulations. A second-order discretization scheme was used for pressure and a second-order upwind scheme was used for momentum, turbulent kinetic energy (k), specific dissipation rate (ω), and species concentrations. The governing equations used to solve each of these variables can be found in the Ansys Fluent theory manual (26). As a transient solver was used, transient formulation was handled using a bounded second-order implicit formulation.

Solutions were first initialized using hybrid initialization and then run for 100 iterations in steady state. This steady-state solution was then used to initialize the transient simulation of the full breathing waveform. A time step of 1E-04 s was used during the full transient simulation. A solution was considered converged when either the residuals dropped 3 orders of magnitude, or 20 iterations were achieved for any given time step. For each simulation, every 0.5 s, data files containing all information at the given time step were saved.

### Computational data

Because the simulations involved no stochastic variability or sampling error, inferential statistical testing was not performed, and a formal sample size calculation was not applicable. Instead, predefined mechanistic contrasts were evaluated by direct comparison of model outputs across conditions.

To measure the effectiveness of the nasal cannula interface during NHF, two metrics were used: CO_2_ volume in the airway and dead-space clearance. The total volume of CO_2_ within the airway was calculated from the volume fraction of CO_2_ in the airway, directly extracted from the model. In this study, dead space in the conducting airways was limited to the nasal cavities and nasopharynx. Dead-space clearance was inferred from the amount of CO_2_ inspired and presented in relative values. To calculate the inspired CO_2_ volume, CO_2_ fraction at the nasopharynx was first used to calculate the flow of CO_2_ at the nasopharynx. This was then integrated over the inspiratory portion of the breathing waveform to give the total inspired volume of CO_2_.

As dead-space clearance is proportional to the volume of inspired CO_2_, a linear equation relating the two was formed. This was undertaken using two reference points at 100% and 0% dead-space clearance. The volume of CO_2_ inspired in the natural breathing, no-therapy case was equated to 0% dead space cleared, and the volume of CO_2_ inspired if no expired air was re-breathed was equated to 100% dead space cleared. Using these two points, a linear equation relating to inspired CO_2_ volume to dead-space clearance was formed. Figures presenting flow patterns and CO_2_ volumes at different time-stamps were extracted from the CFD models also. Other data, such as flow from the prong of the AI and clearance of expired CO_2_ from the nares, was also extracted from the CFD models.

## Results

### Asymmetrical nasal high flow

**FIGURE 2:**
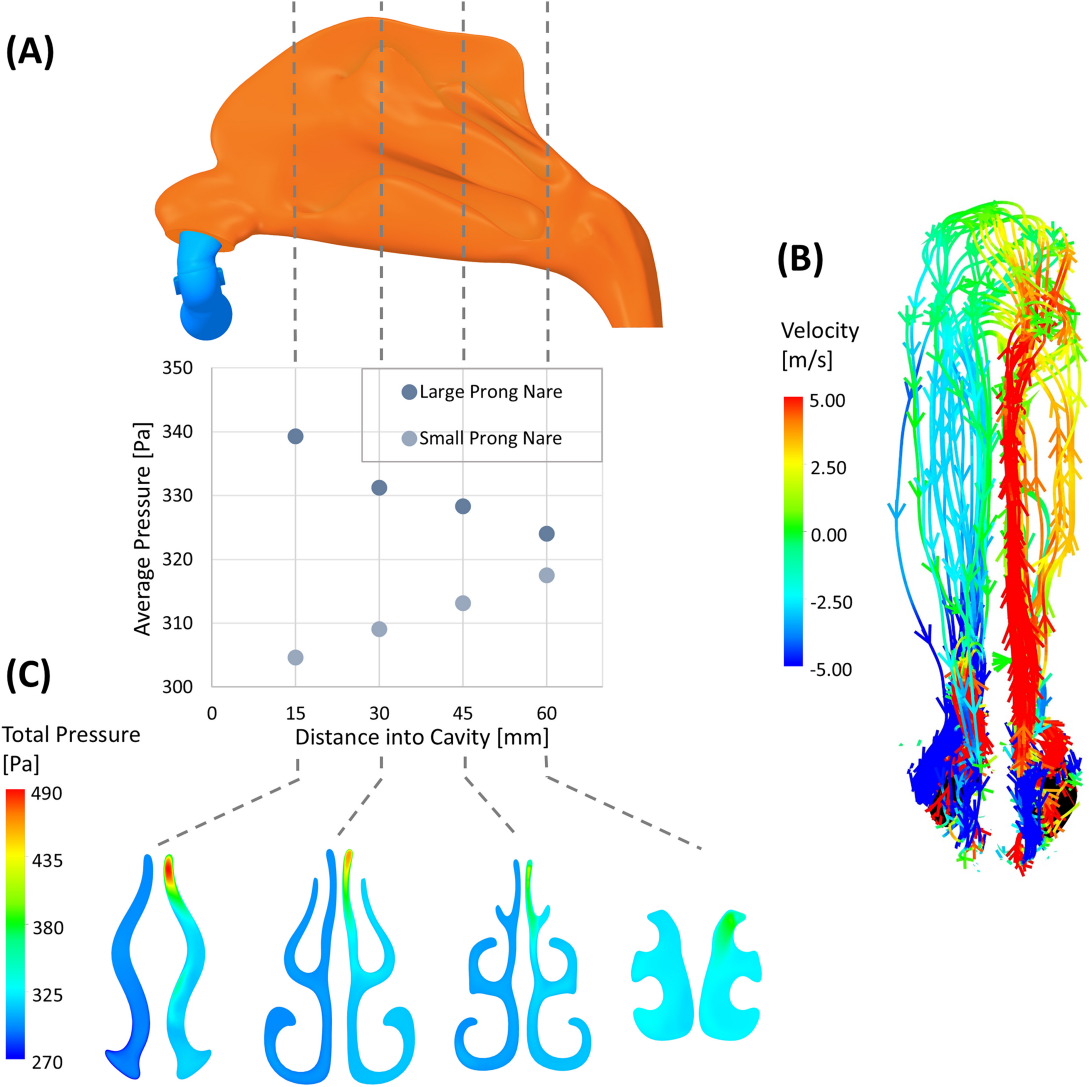
**(A)** Average total pressure at different nasal cavity cross-sections with NHF 60 L/min and an asymmetrical interface (AI) at the end of expiration. **(B)** Streamlines of velocity indicate reverse flow via the choana caused by the pressure difference. **(C)** Pressure contours at 15, 30, 45 and 60 mm into the nasal cavity at the end of expiration.

The effect of asymmetrical NHF at a rate of 60 L/min on airway pressure at the end of expiration is shown in Figure 2. The AI generates a pressure difference between the nasal cavities that is greatest in the superior meatus and decreases toward the choanae. This pressure difference induces reverse flow through the nasal cavity occluded by the smaller prong. Velocity streamlines illustrate this reverse flow toward the choanae, with maximum velocities reaching 5.12 m/s in the inferior nasal meatus.

**FIGURE 3:**
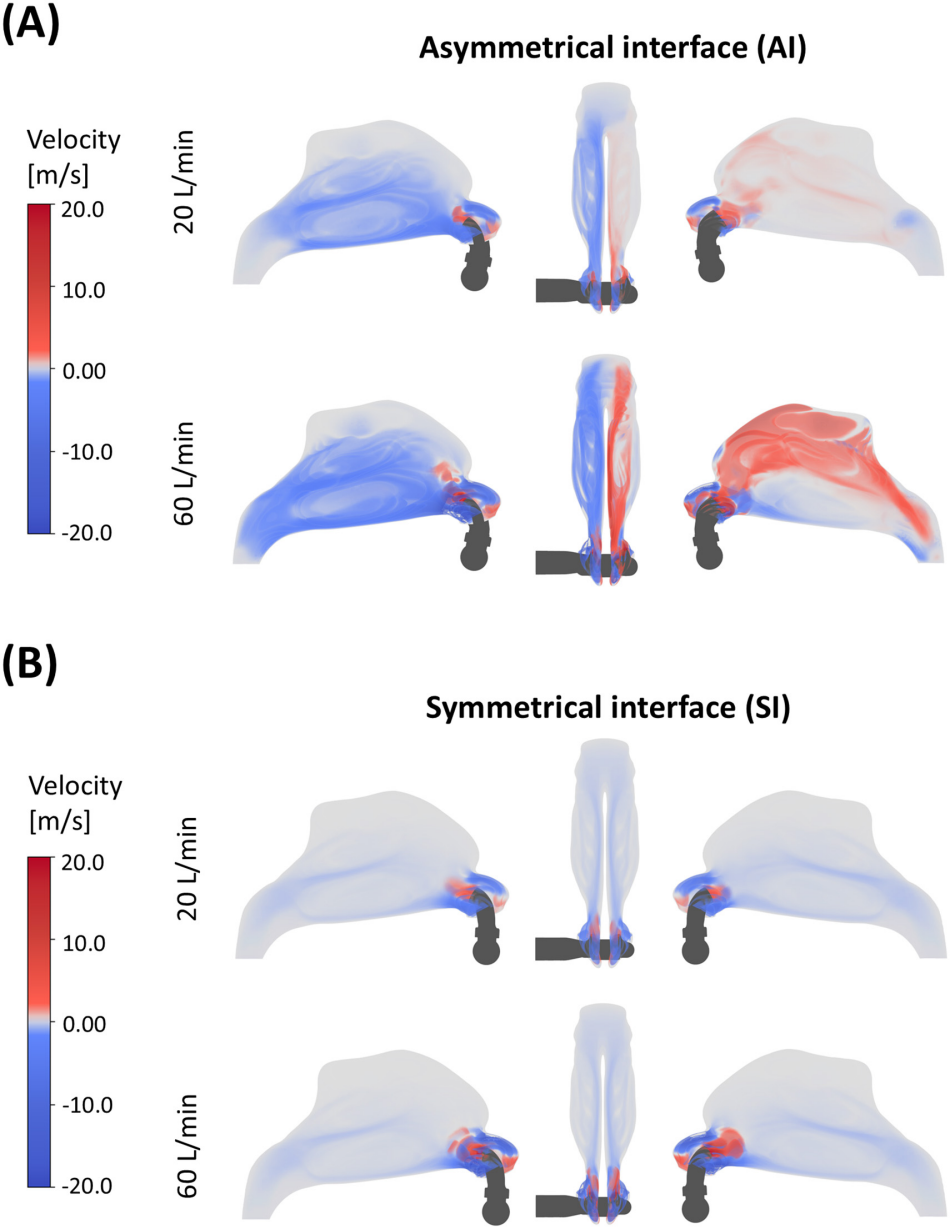
Component of velocity normal to coronal plane in the upper airways at 1.5 s from the start of expiration at a respiratory rate of 15 min^–1^ during nasal high flow (NHF) rates of 20 and 60 L/min with **(A)** asymmetrical interfaces (AI) at the top and **(B)** symmetrical interfaces (SI) interfaces at the bottom.

The component of velocity normal to the coronal plane at mid-expiration for the AI and SI at NHF rates of 20 and 60 L/min is shown in Figure 3. Greater velocity differences are observed with AI, where the positive velocity component (red), associated with the larger prong, is higher in the middle and superior meatuses, while the negative velocity component (blue), associated with the smaller prong, is higher in the middle and inferior meatuses.

**FIGURE 4:**
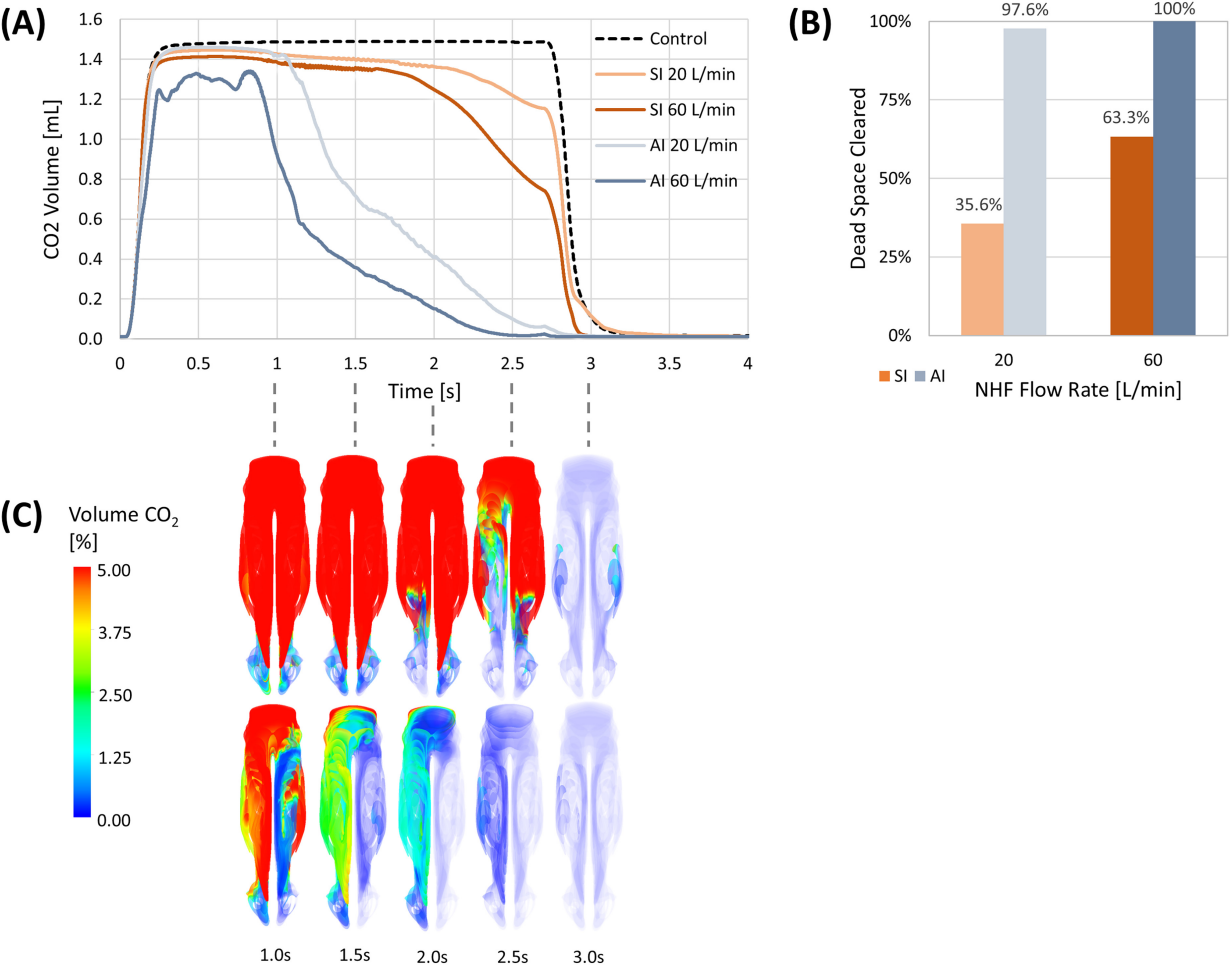
**(A)** Total CO_2_ volume in the airway during a breathing cycle at a respiratory rate of 15 min^–1^ during nasal high flow (NHF) rates of 20 and 60 L/min with asymmetrical interfaces (AI) and symmetrical interfaces (SI). **(B)** Dead-space clearance by NHF at rates of 20 and 60 L/min with the AI and SI during a respiratory rate of 15 min^–1^. **(C)** Volume fraction of CO_2_ during NHF at a rate of 60 L/min with the SI (top) and AI (bottom) from time points of 1.0 to 3.0 s.

The total CO_2_ volume within the nasal cavity during NHF with the AI and SI at a respiratory rate of 15 min^–1^ is shown in Figure 4. Conditions included NHF at rates of 20 and 60 L/min, as well as no therapy as a control. At the onset of expiration, CO_2_-rich gas enters the airway at the nasopharyngeal boundary, representing expired gas from the lungs. A rapid increase in CO_2_ volume occurs during the first 0.5 s of expiration. A flow-dependent decrease in CO_2_ volume begins at approximately 1.0 s with the AI, compared with around 2.0 s with the SI. This results in a marked difference in dead-space clearance by the end of expiration (2.7 s), particularly at an NHF rate of 20 L/min. With the AI, dead-space clearance was almost complete at 60 L/min and only 2.4% lower at 20 L/min. In contrast, the SI showed lower clearance, with values of 35.6% and 63.3% at 20 and 60 L/min, respectively. The CO_2_ volume fraction during the breathing cycle at an NHF rate of 60 L/min for the AI and SI is shown in Figure 4C (see also Supplementary Video 1).

**FIGURE 5:**
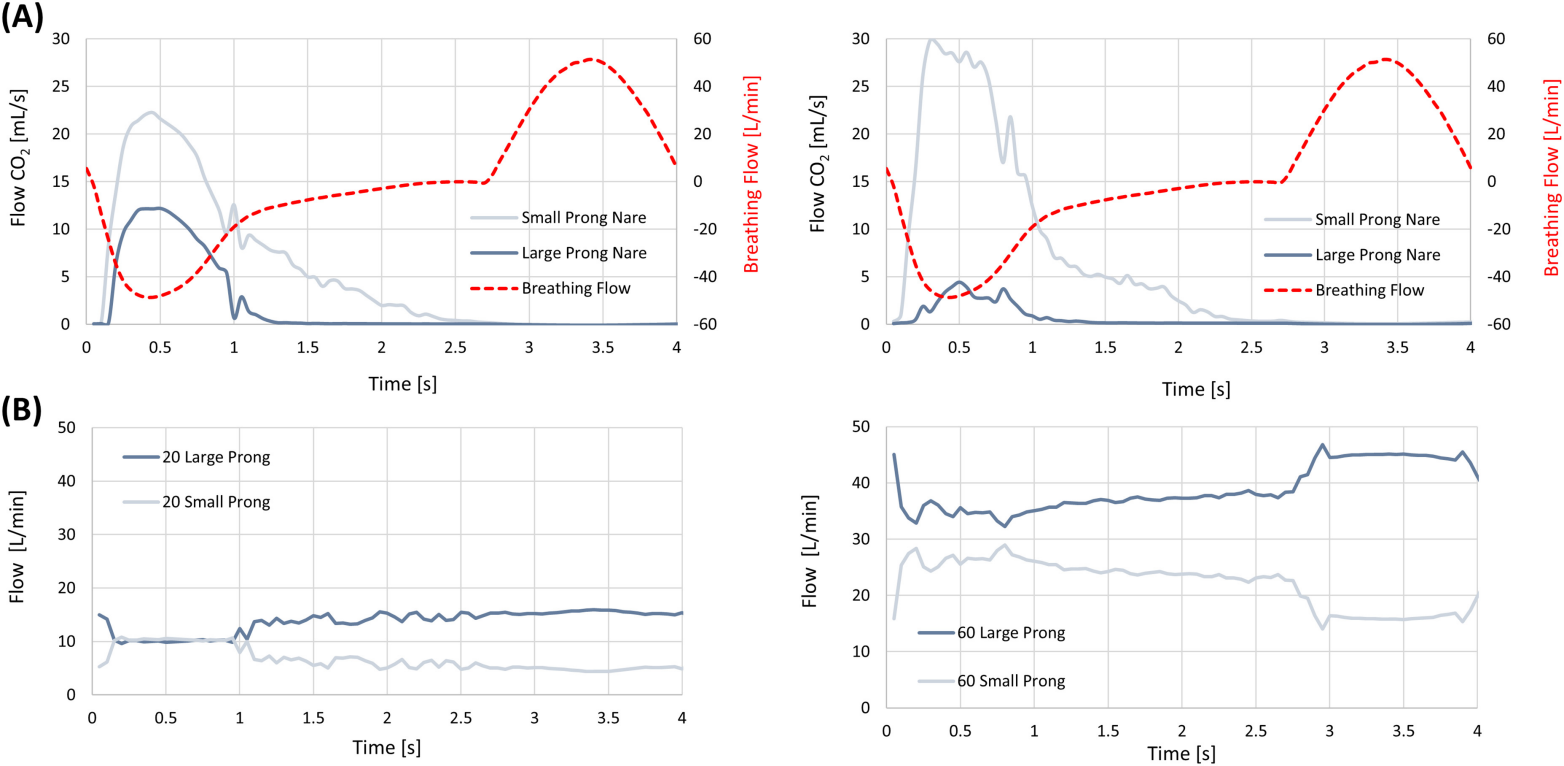
**(A)** Purging of expired CO_2_ measured at the nares during nasal high flow (NHF) at a rate of 20 L/min (left) and 60 L/min (right) with an asymmetrical interface (AI) at a respiratory rate of 15 min^–1^. **(B)** Flow-rate split in the nasal prongs during NHF via an AI at NHF rates of 20 L/min (left) and 60 L/min (right).

Volumetric flow rates of CO_2_ exiting each naris during NHF at rates of 20 and 60 L/min with the AI are shown in Figure 5A. Most CO_2_ is purged through the less occluded naris with the smaller prong. This asymmetry increases at higher NHF rates and is most pronounced near peak expiration. After approximately 1 s, CO_2_ flow through the naris with the larger prong becomes minimal, while flow continues through the opposite naris. Time integration of these flows shows that, at NHF rates of 20 and 60 L/min, 8.2 and 2.6 mL of CO_2_ are purged through the more occluded naris, compared with 21.7 and 26.5 mL through the less occluded naris, respectively.

Figure 5B shows how the rate of NHF splits between the large and small prongs with the AI at 20 and 60 L/min. Throughout the breathing cycle, flow through the larger prong exceeds that through the smaller prong. For both NHF rates, the difference in prong flow is greater during inspiration and smaller during expiration.

### Effect of elevated respiratory rate and NG tube

**FIGURE 6:**
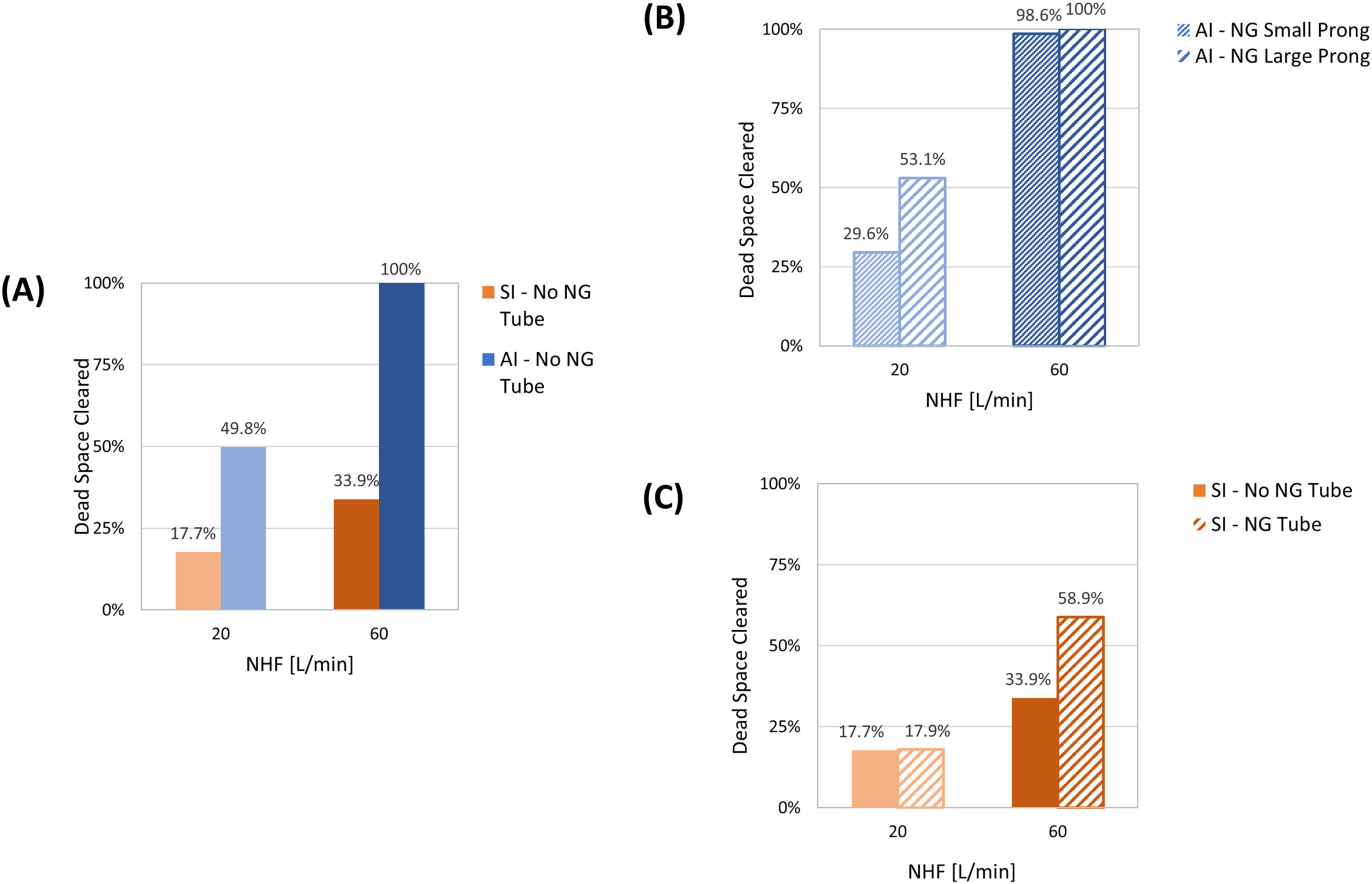
**(A)** Dead-space clearance during a breathing cycle at a respiratory rate of 35 min^–1^ and nasal high flow (NHF) at rates of 20 and 60 L/min with a symmetrical interface (SI) and an asymmetrical interface (AI). No NHF served as a control. **(B)** Dead-space clearance at a respiratory rate of 35 min^–1^ breathing pattern during NHF and AI with NG tube placed on the side of either a small or large prong. **(C)** Dead-space clearance at a respiratory rate of 35 min^–1^ breathing pattern during NHF and SI with a nasogastric (NG) tube.

Dead-space clearance at an elevated respiratory rate of 35 min^–1^ for both the SI and AI is shown in Figure 6A, with corresponding CO_2_ volume changes presented in Supplementary Figure 1. At this elevated rate, NHF with the AI demonstrated substantially greater dead-space clearance than NHF with the SI, increasing from 17.7% to 49.8% at 20 L/min and from 33.9% to complete clearance at 60 L/min.

**FIGURE 7:**
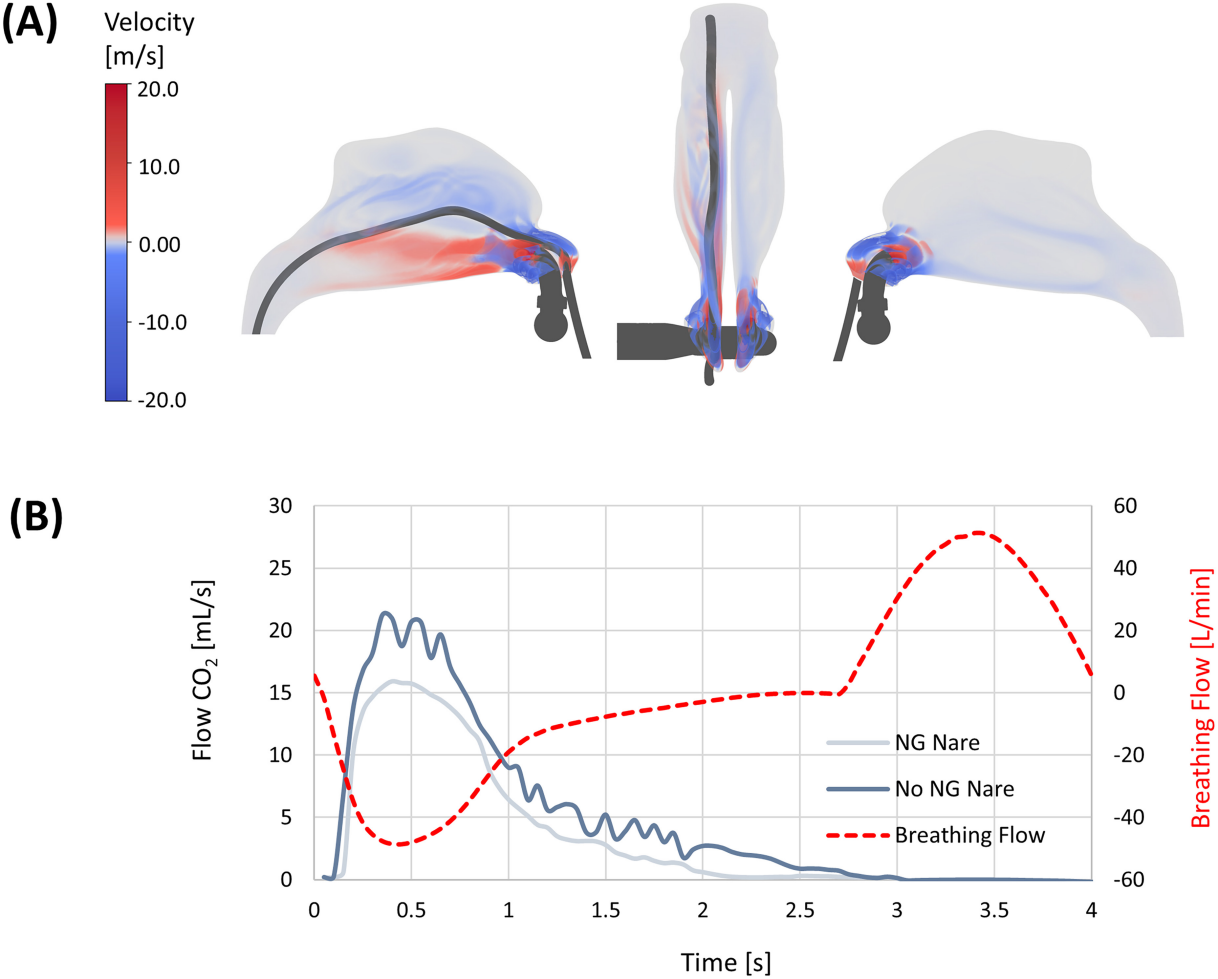
**(A)** Component of velocity normal to the coronal plane in the upper airways at end expiration at a respiratory rate of 15 min^–1^ during nasal high flow (NHF) at a rate of 60 L/min with a symmetrical interface (SI) and a nasogastric (NG) tube, top. **(B)** Purging of expired CO_2_ measured at the nares during an NHF rate of 60 L/min with an SI and an NG tube, at a respiratory rate of 15 min^–1^.

With the SI at a respiratory rate of 35 min^–1^, placement of an NG tube in one naris had little effect on clearance at 20 L/min (17.9% vs. 17.7%). An effect was observed only at 60 L/min, where clearance increased from 33.9% to 58.9% (Figure 6B). With the AI, placement of the NG tube alongside the larger prong increased clearance slightly, at 20 L/min (53.1% vs. 49.8%), whereas placement alongside the smaller prong reduced clearance substantially (29.6% vs. 49.8%) (Figure 6C). At 60 L/min with the AI, near-complete clearance was achieved regardless of NG tube position. Figure 7 illustrates how an NG tube affects gas velocity in the nasal cavities and CO_2_ flow during an NHF rate of 60 L/min with the SI at a respiratory rate of 15 min^–1^. The flow of expired CO_2_ from the naris not occluded by the NG tube exceeds that from the naris containing the NG tube until the end of expiration.

**FIGURE 8:**
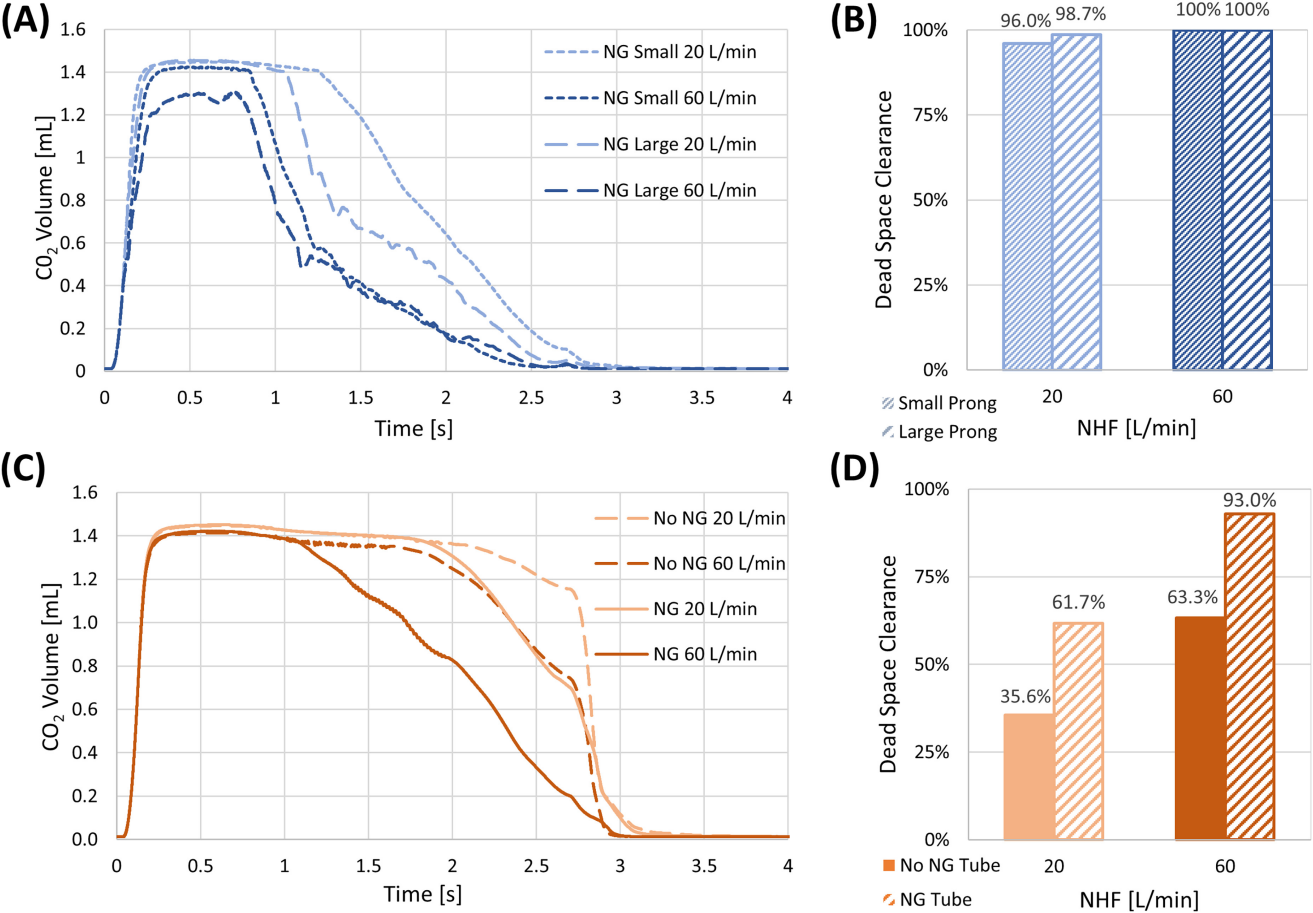
**(A)** Volume of CO_2_ in the upper airway during nasal high flow (NHF) rates of 20 and 60 L/min with an asymmetrical interface (AI) during a breathing cycle at a respiratory rate of 15 min^–1^ with a nasogastric (NG) tube placed on the side of small and large prongs. **(B)** Dead-space clearance by NHF with the NG tube placed on the side of small and large prongs of AI. **(C)** Total CO_2_ volume in the upper airway during NHF with a symmetrical interface (SI) at the same respiratory rate with and without an NG tube. **(D)** Dead-space clearance by NHF with the SI with and without an NG tube.

Figure 8 presents the effect of an NG tube on CO_2_ volume and clearance in the nasal cavity during NHF with the AI and SI at a respiratory rate of 15 min^–1^. For the AI, the NG tube was placed either alongside the smaller or larger prong (Figures 8A,B), whereas the or SI, the effect of the NG tube was compared with no NG tube (Figures 8C,D). With AI, placing the NG tube in the naris occluded by the larger prong led to accelerated reduction of expired CO_2_. However, the effect on final dead-space clearance was minor, as both cases reached nearly 100% clearance (Figures 8A,B).

With the SI, the NG tube increased dead-space clearance at an NHF rate of 20 L/min from 35.6% to 61.7%, and at 60 L/min from 63.3% to 93%. The NG tube also increased airway pressure during NHF. With the AI, the highest pressure values were observed when the NG tube was placed in the naris alongside the smaller prong (see Supplementary Figure 2 online).

## Discussion

Computational fluid dynamics simulations revealed that asymmetry in gas flow within the upper airways, caused by either an AI or an NG tube during NHF, could increase dead-space clearance. Different prong sizes in the AI affect gas flow both inside and outside of the prongs. In contrast, the NG tube with the SI primarily alters expiratory flow and reduces the re-breathing volume of expired gas on the side of the nasal cavity where it is placed.

An increase in the respiratory rate to 35 min^–1^, which is common in patients with ARF, shortens the breathing cycle and results in inspiration and expiration times of approximately equal duration. This reduces the efficiency of dead-space clearance due to the limited time available for purging expired gas (8). Both the AI and the NG tube used with the SI increased dead-space clearance during NHF at this higher breathing frequency. However, NHF with the SI and an NG increased clearance only at an NHF rate of 60 L/min, which can be explained by the higher differential pressure between the nasal cavities at a higher NHF rate.

Positioning the NG tube on the side of the nose with the larger AI prong improves clearance by increasing air-flow asymmetry, although this could only produce a noticeable physiological effect at lower NHF settings, such as 20 L/min. At a low breathing frequency of 15 min^–1^, which could be observed in stable patients, the upper airway is almost completely cleared of expired gas by NHF with the AI, making the relative position of the NG tube with respect to the large or small prong less significant.

Nasal high flow delivered with the AI splits flow both inside and outside the prongs, generating a pressure difference between the nasal cavities that leads to reversed flow via the choana, thereby reducing the volume of expired gas that is subsequently re-breathed. Within the AI, flow inside the prongs is biased toward the larger prong, with this difference increasing during inspiration. In contrast, flow outside the prongs is biased toward the side occluded by the smaller prong, with this component increasing toward the peak of expiration. This phenomenon was previously predicted in a mathematical model and was attributed to differences in flow resistances (15). Increasing NHF rates (60 L/min) further amplify these flow asymmetries.

Velocity streamlines revealed opposite flow directions at the end of expiration, demonstrating the mechanism responsible for increased clearance and reduced re-breathing during NHF with the AI. The increased velocity of gas from the larger prong in the upper and middle meatuses can be explained by prong positioning and the smaller cross-sectional area of these regions. After reversal of flow through the choanae, the maximum velocity occurs in the middle and inferior meatuses, possibly due to lower flow resistance on the right side of the nasal cavity, where the naris is occluded by the smaller prong. NHF with the AI almost completely cleared expired gas at a breathing rate of 15 min^–1^ at both 20 and 60 L/min, substantially outperforming the SI. Analysis of expired CO_2_ volume fractions showed that clearance with the AI begins early during expiration (Figure 4). This explains the significantly higher dead-space clearance achieved with the AI at a respiratory rate of 35 min^–1^, when clearance time is limited and efficiency with the SI is markedly reduced (Figure 6). An NG tube also introduces asymmetry in upper-airway flow during NHF with both the AI and SI. By unilaterally increasing occlusion, the NG tube reduces flow on one side of the nasal cavity and decreases re-breathing. During NHF with the AI, the preferred NG tube position is alongside the larger prong, which increases asymmetry and pressure differences. However, with the AI, this effect is substantial only at high breathing frequencies and low NHF rates – conditions that are unlikely in clinical practice, as tachypneic patients often require higher positive airway pressure. Simulations suggest that other sources of asymmetry could similarly affect clearance. Placement of the NG tube alongside the smaller prong reduced occlusion asymmetry but slightly increased nasopharyngeal pressure during expiration, indicating increased overall dynamic resistance. Larger NG tubes commonly used in clinical practice could further amplify these effects. In bench-top experiments, Vieira et al. concluded that small NG tubes have minimal impact, whereas larger tubes could produce significant physiological effects. In addition, deformation of one of the prongs by the NG tube could substantially impair the clearance of expired gas (21).

Consistent with previous studies, the NHF rate influences expired-gas clearance in the upper airways (9, 14, 15, 24). This effect on dead-space clearance was most prominent at elevated respiratory rates, where shortened breathing cycles limit clearance time. Van Hove et al. reported an exponential decrease in inspired CO_2_ volume with increasing NHF rates using a conventional SI (24). Direct comparison with the present study is limited due to differences in airway extent and breathing pattern. However, inspired CO_2_ volume with the AI also decreased non-linearly with increasing NHF rates, particularly at a respiratory rate of 35 min^–1^. At 15 min^–1^, an asymptotic minimum appeared to be reached at an NHF rate of 20 L/min. For the SI, inspired CO_2_ volume decreased non-linearly as well, but with higher asymptotic value than with the AI. Differences from Van Hove et al. could be attributed to the shallow breathing pattern used in the present simulations, which reduced resistance to cannula flow.

Increased dead-space clearance with the AI compared with the SI has been demonstrated in bench-top experiments too, which showed that the AI generates reverse flow through the choanae (15). Adding the NG tube further modifies the kinetics of gas in the upper airways, unilaterally changing the nare occlusion, resulting in higher airway pressure and improved clearance (21). Differences in CO_2_ concentration between that study and the present work can be attributed to differing levels of nare occlusion. Overall, these findings support the conclusion that increased asymmetry in the nasal cavities could enhance dead-space clearance during NHF.

The CFD methodology used in this current study aligns with similar studies in the literature that also modeled NHF in the upper airway using CFD (23, 24, 27, 28). A study by Kacinski et al. focused on the validation of CFD modeling in the context of NHF therapy in the upper airway (25). They examined mesh sizing, flow modeling approaches, and discretization schemes. A model investigated by the authors, which closely matched the model used in this study, was found to have only a 1.65% error in the fraction of CO_2_ at the airway inlet compared to experimental data.

Schillaci et al. investigated the importance of numerical schemes, flow models, and domain truncation when modeling air-flow within the upper airway using CFD (29). They found that truncation of the computational domain at the nasopharynx does not fully capture the laryngeal jet during expiration. In that study, expiratory flow was modeled as uniform, and as a result the laryngeal jet was not captured. The authors also found the Reynolds-Averaged Navier-Stokes (RANS) flow model to be less accurate than Large Eddy Simulations (LES). RANS was found to overestimate the pressure drop from inlet to outlet by 2–4 Pa. LES can be up to 60 times more computationally expensive than RANS and therefore was not feasible to use in this current study. They concluded that the order of the numerical scheme had the largest impact on solution accuracy. First-order discretization created a difference of up to 50% in global values compared to second-order schemes. A second-order accurate scheme was used in the current simulation.

### Limitations

In this study, the interior of the AI cannula itself was included in the computational model. This was necessary to resolve the flow within the AI to capture the flow rate split between the two differently-sized prongs. Other NHF computational CFD studies have only investigated the SI and modeled the flow-rate split between the prongs as 50-50, as was undertaken for NHF with the SI in the present study. However, the flow-rate split in the SI could not actually be ideally equal between the prongs, as the gas is supplied to one side of the cannula. This results in asymmetry, with the flow streamline being directed to the distal prong, causing a slightly higher flow from this prong. A bench-top study with a simplified optical model of the upper airways found that the SI resulted in some asymmetry, but this was minor compared to the flow-rate split found with the AI, which has different internal cross-sectional areas for the nasal prongs (15). Modeling the 50-50 prong flow-rate split allowed exploration of the fundamental effect of symmetrical NHF while maintaining consistency of comparison with previous studies.

Only one NG tube size, 8 Fr (1 Fr = 1/3 mm), was used in the model. This relatively small size was selected because it fits within the naris occluded by the large prong of AI without deforming the prong. In adult patients, larger NG tubes are commonly used and would further increase asymmetry between the nares. Trends from the present work suggest that a further increase in asymmetry could result in even greater clearance; however, additional studies are required to confirm this.

A larger NG tube could cause deformation of the prong as well, which could increase the resistance of the deformed prong and thereby divert more flow through the undeformed prong. This effect would compete with the increased occlusion introduced by the NG tube itself in determining overall flow asymmetry. Future studies could quantify the extent to which prong deformation contributes to asymmetry and, consequently, clearance. It could also be of interest to investigate whether the effectiveness of the AI during NHF could be replicated solely by inducing greater asymmetric resistance using an SI. The present findings suggest that this could not be possible; however, identifying an optimal level of resistance or occlusion could have clinical relevance.

The NG tube could also affect the physiology of the velopharyngeal valve and potentially influence leakage of expired gas through an open mouth during NHF. Anatomical structures below the nasopharynx were not investigated, and the position of the soft palate and the open or closed mouth during NHF could need to be addressed in future studies.

Other potential sources of asymmetry in the airway include the physiological nasal cycle, nasal septum deviation, or other anatomical or disease-related abnormalities, such as turbinate hypertrophy (30, 31). The nasal cycle is a normal physiological phenomenon involving periodic congestion and decongestion of the nasal cavities, which is often asymmetrical and alternates from one side to the other every few hours. Because the prongs of the cannula used during NHF do not reach the internal nasal valve area, changes in nasal congestion are unlikely to alter the occlusion in the nasal vestibule produced by the short prongs. Periodic swelling in the nasal passages could therefore have different effects from the modeled unilateral occlusion caused by an NG tube. The impact of the nasal cycle, deviated nasal septum, turbinate hypertrophy, polyps or other changes in the nasal cavity that could affect air-flow and dead-space clearance during NHF should be further investigated to evaluate the effectiveness of the therapy.

The airway geometry used in the model was derived from averaged geometries of a European population. It is well established that upper airway anatomy varies across ethnicities. Although this limits the direct generalizability of the results to the entire adult population, the findings still demonstrate the effect of flow-path asymmetry within the upper airway. The use of an averaged geometry also resulted in a smoother and more regularized anatomy than is typical of an individual airway, which could exaggerate the observed effects. Both ethnicity and geometric averaging could be addressed in future studies by investigating a range of individual anatomies across different ethnicities, ages and genders. In addition, the shape and size of the cannula could vary, and the NHF interface could consist of a single prong or a combination of prongs with a mask. All these factors, as well as the positioning of nasal prongs and an NG tube in the nares, could influence airway pressure and clearance of expired gas during NHF.

Computational fluid dynamics simulations were performed under steady NHF, but variable flow from the cannula during a breathing cycle could impact both pressure and clearance as well. Also, beyond increased respiratory rate, the breathing patterns of patients with COPD, who experience expiratory flow limitation and hyperinflation, could reduce the efficiency of dead-space clearance by NHF (15). These breathing patterns were not simulated, but in patients with bronchoconstriction, expired CO_2_ sharply rises toward the end of expiration, often resulting in a capnogram waveform referred to as a “shark fin” shape (30). This expiratory CO_2_ profile should be modeled too, as NHF would preferentially clear the most CO_2_-rich portion of expired gas.

The simulations assumed gas was heated to 37 °C with 100% relative humidity. It is well established that mucociliary transport in the airway epithelium can be rapidly impaired by gas with reduced humidity (32). Asymmetric flow in the upper airway during NHF could further exacerbate these effects by reducing heat and moisture exchange in the upper airways, and this warrants further investigation.

Another limitation of this study is the absence of direct validation of the CFD model against experimental or clinical data. To mitigate this, relevant validation studies and models from existing literature were reviewed and replicated where appropriate. However, this approach does not replace validation using real-world data and remains a significant limitation to the current work.

The project was conducted through a collaboration between Fisher & Paykel Healthcare and the University of Canterbury. In accordance with University of Canterbury policy, the scholarship agreement guaranteed full academic independence, including freedom in study design, data analysis, interpretation and publication. Comparisons were restricted to direct mechanistic contrasts, and all outcomes were derived objectively from solver-generated flow and gas-concentration fields, without *post hoc* adjustment or selective reporting. However, the possibility of residual bias cannot be entirely excluded, and the findings should therefore be interpreted as hypothesis-generating mechanistic evidence rather than proof of clinical superiority or patient benefit.

### Clinical significance

The CFD simulations should not be directly extrapolated to clinical settings, as they were primarily designed to elucidate the mechanisms of dead-space clearance by NHF. The study reveals the role of asymmetry achieved by the AI and/or an NG tube during NHF in clearing expired CO_2_ from the upper airway, leading to a reduction in re-breathing and decreasing dead space effectively. Patients with increased dead-space ventilation, such as those with ARF who have increased physiological dead space and reduced alveolar volume, would benefit from even a relatively small reduction in dead space, which can be improved by the AI (17).

These patients frequently exhibit an increased respiratory rate, which could reduce clearance efficiency, and often have an NG tube post-extubation. The present study indicates that an NG tube, provided it does not deform the cannula prong, could also enhance dead-space clearance efficiency. Yet, a higher level of clearance is achieved with the AI, which produces a substantial split in NHF at the end of expiration even at low flow rates. The simulations suggest a minor effect of a small-diameter NG tube position relative to the small or large prong, with a slight improvement in clearance when the NG tube is placed alongside the larger prong, thereby increasing asymmetry in naris occlusion and reverse flow in the choana.

The combined use of NHF and an NG tube should be approached with caution, as the larger-sized tube, commonly used post-extubation, could substantially occlude the naris, particularly when it has a large outside diameter and is placed alongside the smaller prong, potentially leading to near-complete airway occlusion, deformation of a prong and reduced tolerability of the therapy. This study had a mechanistic focus; the simulations were performed in the strictly controlled conditions and did not address optimal sizing of cannula interfaces, which remains an important topic for future clinical investigation.

In addition to NG tubes, nasoduodenal tubes are commonly used for enteral nutrition in children with bronchiolitis, a population in which NHF is increasingly used (33). Data from the current *in silico* study indicates that feeding tubes could influence the physiological effects during NHF in young children too, as these tubes may unilaterally increase occlusion area in the upper airways as well (34, 35).

## Conclusion

A numerical fluid dynamics study of the upper airway during NHF mechanically demonstrated a beneficial role of asymmetry in the upper airways in clearing anatomical dead space. During NHF with the AI, asymmetrical naris occlusion reduces expired flow on one side and increasing it on the opposite side, resulting in reverse flow via the choana and reduced re-breathing from the upper airways. NHF with the AI contrasts with NHF combined with the SI and NG tube, as only the latter requires higher NHF rates to enhance clearance, introducing differential pressure in the upper airway. Occluding the naris by an NG tube on the side with the large prong in the AI amplifies flow asymmetry, improving dead-space clearance by NHF provided that no deformation of a prong in the cannula interface is produced.

## Data Availability

The raw data supporting the conclusions of this article will be made available by the authors, without undue reservation.

## Supplementary material

Supplementary Figure 1 Changes in CO_2_ volume in the upper airway during a breathing cycle at a respiratory rate of 35 min^−1^ with nasal high flow (NHF) of 20 L/min and 60 L/min using a symmetrical interface (SI), asymmetrical interface (AI), and nasogastric (NG) tube. **(A)** SI and AI without an NG tube. **(B)** SI with an NG tube. **(C)** AI with an NG tube.

Supplementary Figure 2 Changes in airway pressure during nasal high flow (NHF) at 60 L/min with an asymmetrical interface (AI) and a nasogastric (NG) tube placed in the naris alongside either the smaller or larger prong. **(A)** During a breathing cycle at a respiratory rate of 15 min^−1^. **(B)** During a breathing cycle at a respiratory rate of 35 min^−1^. The figure demonstrates that the highest pressure during NHF is achieved when the NG tube is placed in the naris alongside the smaller prong of the AI, despite a similar combined occlusion area.

Supplementary Video 1 The video demonstrates the distribution of expired CO_2_ modelled in the upper airway during a breathing cycle at a respiratory rate of 15 min^−1^ and nasal high flow of 60 L/min with symmetrical and asymmetrical interfaces.
