# Supplementary figures and images for: Asymmetry from an asymmetrical cannula interface and nasogastric tube during nasal high flow enhances dead-space clearance: a fluid dynamics study
Source: Front Med (Lausanne). 2026 May 29;13:1823238. doi: 10.3389/fmed.2026.1823238 (PMC13260638; doi:10.3389/fmed.2026.1823238)

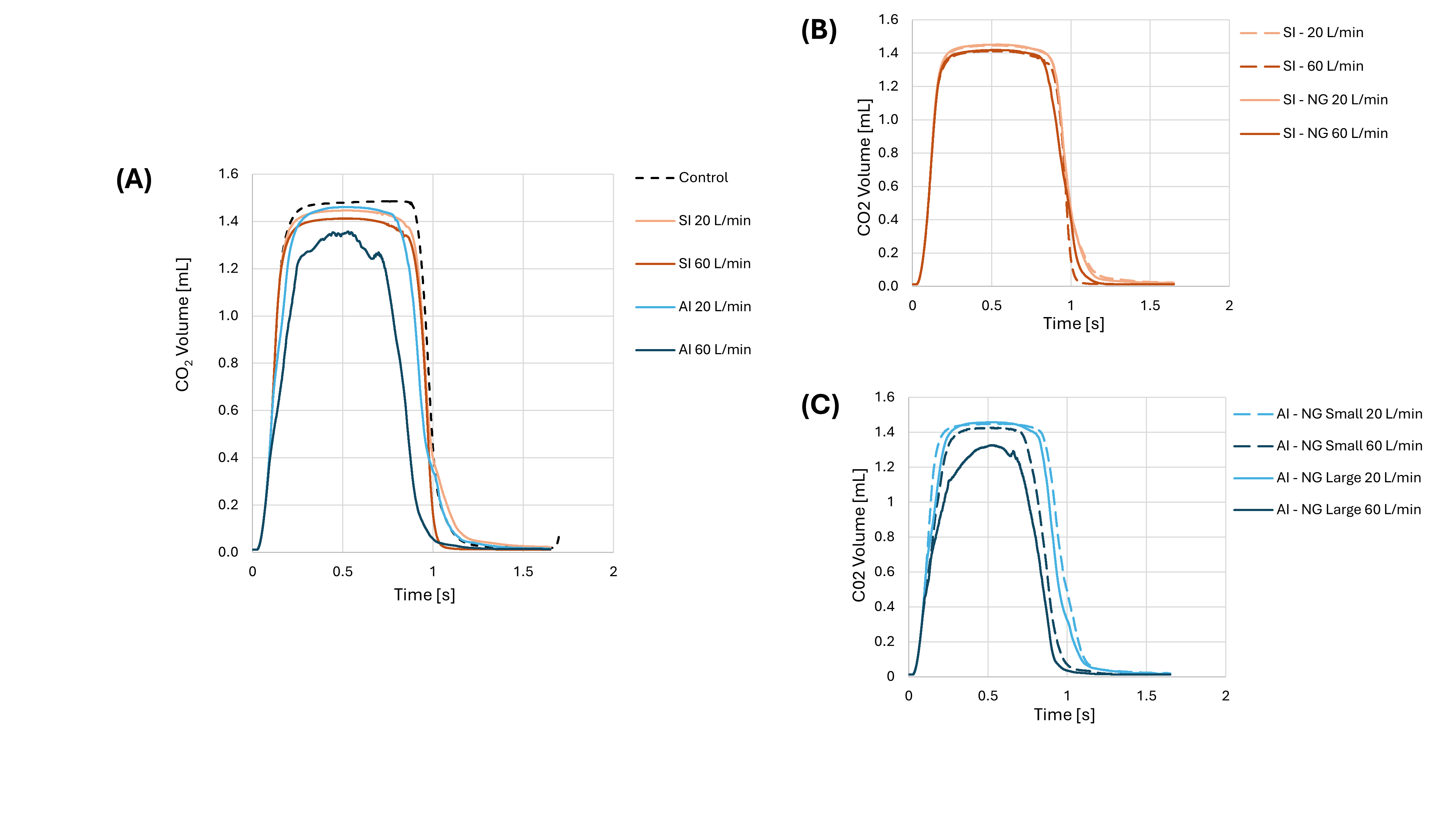

Supplement: Supplementary Figure 1 — Changes in CO2 volume in the upper airway during a breathing cycle at a respiratory rate of 35 min−1 with nasal high flow (NHF) of 20 L/min and 60 L/min using a symmetrical interface (SI), asymmetrical interface (AI), and nasogastric (NG) tube. (A) SI and AI without an NG tube. (B) SI with an NG tube. (C) AI with an NG tube. [file Image_1.tif]

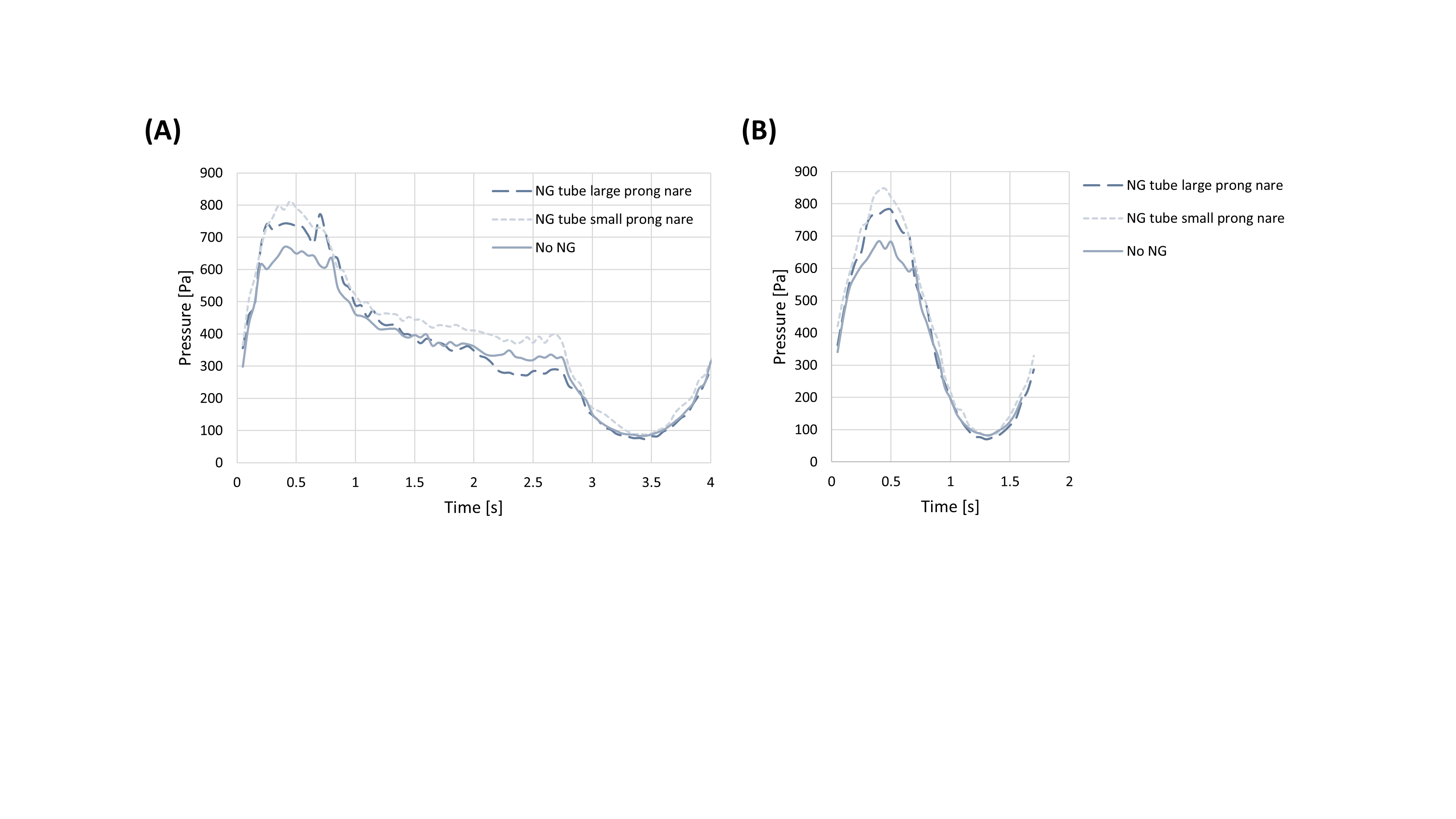

Supplement: Supplementary Figure 2 — Changes in airway pressure during nasal high flow (NHF) at 60 L/min with an asymmetrical interface (AI) and a nasogastric (NG) tube placed in the naris alongside either the smaller or larger prong. (A) During a breathing cycle at a respiratory rate of 15 min−1. (B) During a breathing cycle at a respiratory rate of 35 min−1. The figure demonstrates that the highest pressure during NHF is achieved when the NG tube is placed in the naris alongside the smaller prong of the AI, despite a similar combined occlusion area. [file Image_2.tif]
